# Bibliometric Analysis of Publications on the Omicron Variant from 2020 to 2022 in the Scopus Database Using R and VOSviewer

**DOI:** 10.3390/ijerph191912407

**Published:** 2022-09-29

**Authors:** Hasan Ejaz, Hafiz Muhammad Zeeshan, Fahad Ahmad, Syed Nasir Abbas Bukhari, Naeem Anwar, Awadh Alanazi, Ashina Sadiq, Kashaf Junaid, Muhammad Atif, Khalid Omer Abdalla Abosalif, Abid Iqbal, Manhal Ahmed Hamza, Sonia Younas

**Affiliations:** 1 Department of Clinical Laboratory Sciences, College of Applied Medical Sciences, Jouf University, Sakaka 72388, Saudi Arabia; 2 Department of Computer Sciences, National College of Business Administration and Economics, Lahore 54700, Punjab, Pakistan; 3 Department of Basic Sciences, Deanship of Common First Year, Jouf University, Sakaka 72388, Saudi Arabia; 4 Department of Pharmaceutical Chemistry, College of Pharmacy, Jouf University, Sakaka 72388, Saudi Arabia; 5 Allied Health Department, College of Health and Sport Sciences, University of Bahrain, Zallaq 32038, Bahrain; 6 Department of Computer Science, Lahore Leads University, Lahore 54000, Punjab, Pakistan; 7 School of Biological and Behavioural Sciences, Queen Mary University of London, London E1 4NS, UK; 8 Prince Sultan University, Riyadh 11586, Saudi Arabia; 9 Department of Medical Microbiology, Faculty of Medical Laboratory Sciences, Omdurman Islamic University, Omdurman 14415, Sudan; 10 HKU-Pasteur Research Pole, School of Public Health, LKS Faculty of Medicine, The University of Hong Kong, Hong Kong, China

**Keywords:** bibliometrics, Omicron variant, Scopus, Biblioshiny application, Bibliometrix package, R, VOSviewer

## Abstract

Human respiratory infections caused by coronaviruses can range from mild to deadly. Although there are numerous studies on coronavirus disease 2019 (COVID-19), few have been published on its Omicron variant. In order to remedy this deficiency, this study undertook a bibliometric analysis of the publishing patterns of studies on the Omicron variant and identified hotspots. Automated transportation, environmental protection, improved healthcare, innovation in banking, and smart homes are just a few areas where machine learning has found use in tackling complicated problems. The sophisticated Scopus database was queried for papers with the term “Omicron” in the title published between January 2020 and June 2022. Microsoft Excel 365, VOSviewer, Bibliometrix, and Biblioshiny from R were used for a statistical analysis of the publications. Over the study period, 1917 relevant publications were found in the Scopus database. Viruses was the most popular in publications for Omicron variant research, with 150 papers published, while Cell was the most cited source. The bibliometric analysis determined the most productive nations, with USA leading the list with the highest number of publications (344) and the highest level of international collaboration on the Omicron variant. This study highlights scientific advances and scholarly collaboration trends and serves as a model for demonstrating global trends in Omicron variant research. It can aid policymakers and medical researchers to fully grasp the current status of research on the Omicron variant. It also provides normative data on the Omicron variant for visualization, study, and application.

## 1. Introduction

On 24 November 2021, the World Health Organization (WHO) announced Omicron as a COVID-19 variant of concern, leading to travel restrictions, a scramble to speed up booster immunization programs, and new attempts to address vaccine inequity [1]. On 29 November 2021, the WHO declared Omicron a “very high” threat worldwide, and preliminary research indicated that it might be more transmissible than other variants, potentially leading to infection outbreaks [2]. Omicron has quickly surpassed Delta (B.1.617.2) as the predominant type of SARS-CoV-2 spreading worldwide. The Omicron spike (S) protein consists of 30 mutations and constitutes the most immune-evasive variant of concern discovered thus far, outperforming Beta (B.1.351) through its capacity to withstand neutralization by antibodies (Abs) [3]. Compared to the Delta variation, Omicron has gained much attention. An in-silico investigation revealed that the pathogenicity of Omicron might be more than tenfold that of the original virus and about two times that of the Delta variant [4]. According to published statistics, experts estimate that the transmission rate of Omicron is 1.4 to 3.1 times more than that of Delta.

As of 26 November 2021, travel-related incidents had also been reported in Hong Kong, Belgium, and Israel. Several cases of VOC Omicron have been reported in Austria, Belgium, Australia, Canada, Denmark, the Czech Republic, Germany, France, Italy, the Netherlands, and also the United Kingdom on 29 November 2021, three days just after the WHO notification [5]. According to reported data, the cases of COVID-19 account for fewer than 25% of the actual cases during the Omicron wave. This is most likely due to a large number of infected people who are asymptomatic or have moderate symptoms. Moreover, it shed light on the role played by immunization in pandemic dynamics and revealed a three- to four-fold increase in mortality among the unvaccinated population in these countries [6].

Due to a high number of cases and mortality rate, travel restrictions were imposed on southern African countries by countries such as the United Kingdom, the United States, Gulf Cooperation Council member countries, and European Union member states [7]. Israel and Japan effectively closed their borders to outside visitors. The fear that the variant could weaken the efficiency of COVID-19 vaccines spurred the United Kingdom and the United States to announce a hastening of booster vaccine rollouts for better protection [8]. The United Kingdom also tightened the requirements for PCR tests for anyone entering the country, introduced self-isolation for contacts of suspected Omicron cases, and added 10 Southern African nations to a travel warning list. In the United Kingdom, masking was resumed in stores and public transportation. The viral pandemic has adversely affected societies and economies [9,10,11].

The rise of infectious diseases is regarded as one of the world’s unavoidable public health challenges. However, modern technology and medical research advances may lessen the detrimental impact of infectious diseases. For instance, the major outbreak of a new infectious disease, COVID-19, has posed significant difficulties to the world economy and public well-being [12,13]. In the last two decades, an increasing number of relevant researchers have explored the prognosis of infectious diseases and published numerous publications [14,15]. These publications explored the research trends in infectious disease areas such as Ebola virus disease, dengue, and Middle-East respiratory syndrome coronavirus (MERS) [16,17,18]. Only a few investigations on the characteristics and quality of literature include infectious illness research prediction using bibliometric approaches.

Bibliometrics is a field that evaluates research and scientific advancement on a quantitative and qualitative level. Due to the requirement to evaluate research results at the individual, institutional, and geographic levels, the discipline has gained appeal in recent years. Due to this requirement, many factors are raised, and databases like Scopus, Web of Science, Dimensions, PubMed, and Google Scholar are created to calculate these values. The volume of research papers and other scientific activities has resulted in an enormous amount of data being produced and preserved. Without the use of advanced tools and processes, the analysis of the data is impossible. The employment of ML algorithms to perform operations on these databases including classification, regression, clustering, and relationships becomes inevitable [19,20]. The performance indicators for research make specific use of citation data. Citations have emerged as a crucial factor in assessing the success of authors, articles, and journals. In order to assess the significance of ML in bibliometrics, it is important to consider how ML approaches can be used to estimate the number of citations, offer helpful advice for creating new bibliometric indexes, and uncover relationships between various variables [21,22].

Bibliometrics and visualization have been characterized as critical tools for detecting emerging infectious disease outbreaks and essential techniques for evaluating scientific research [23,24]. This observation is valid in light of the current situation in which large volumes of data are being transferred [23,25,26]. Bibliometrics is also commonly used in various disciplines to assess the quantitative and qualitative aspects of scientific research [27]. A similar study presented a bibliometric analysis and highlighted the connection between the drug and COVID-19, as well as the newly revised information on the monoclonal antibody REGEN-COV from the Food and Drug Administration and other agencies [28,29]. It focused on REGEN-COV’s bibliometric data in PubMed and Google Scholar for the past three years, 2020, 2021, and 2022. A thorough discussion of multicriteria decision analysis in healthcare is provided, along with comprehensive research and bibliometric analysis [30]. Multiple-criteria decision analysis (MCDA) was applied in a range of healthcare contexts, and a diverse range of methodological techniques was used, per the outcomes of this review [31].

This study aimed to perform an bibliometric analysis of publications related to the Omicron variant indexed in the Scopus database. A quantitative approach was used to conduct a bibliometric analysis of published articles to achieve this objective. This study employs Bibliometrix, an R package with a web-based interface, Biblioshiny, and VOSviewer for bibliometric analysis [32,33]. To our knowledge, this is the first bibliometric study to assess trends in Omicron variant-related epidemiological research. By identifying the key research sites for the Omicron variant, the findings of this study can help create national and institutional research plans. Furthermore, the visualization data or evidence can be used to study the historical record of research output in a particular field and identify potential future research paths and collaborative relationships [34,35].

The manuscript’s organization is as follows: Section 1 describes the Omicron variant and provides a literature review for the bibliometric analysis. Section 2 presents the materials and methods. The results are discussed in Section 3, which is followed by Section 4, containing a discussion on the strengths and limitations of the study. Finally, the conclusion and future directions are presented in Section 5 and Section 6.

## 2. Materials and Methods

Bibliometric investigations allow the development of a unique perspective from a reasonably extensive analysis. The bibliometrics utility of the R package is intended for quantitative scientometrics and informetrics [36]. Furthermore, bibliometric technologies allow the categorization and analysis of large amounts of historical data derived from research conducted over a given period in order to retrieve information from the repository. Bibliometric analysis and meta-analysis rely on quantitative techniques and can therefore avoid or mitigate the bias, in contrast to systematic literature reviews that typically rely on qualitative techniques, which may be marred by interpretation bias from scholars with various academic backgrounds [37].

This study used bibliometric analysis to examine recent trends in Omicron variant-based research. Bibliometric analysis is a quantitative statistical evaluation of publications that is objective, rigorous, transparent, and repetitive. Content analysis and descriptive analysis are two of the most critical bibliometric techniques. The descriptive analysis involves the scrutiny of several publications and journal indices that aid in evaluating the publication effectiveness of authors and sources. In contrast, content analysis uncovers the intellectual structures of particular disciplines, typically through keyword and citation studies that identify trending topics, thematic evolution, and research foci.

Various databases exist for importing bibliographic data, such as Scopus, Web of Science (WoS), Dimensions, Cochrane Library, Lens, and PubMed, and each one has unique properties and functions. The Web of Science and Scopus are currently the most widely used literature databases for almost all disciplines [38,39]. In this study, we conducted a document search on the Scopus database, as it can provide a substantial number of papers and offers more citation-rich data [40]. Scopus is an integrated database that allows academics to explore and evaluate publications, patents, clinical trials, and policy papers. We searched publication titles, abstracts, and authors’ keywords. The search criteria were “Omicron” AND “COVID-19” OR “SARS-CoV-2” [41]. The subject areas that were included in this study were biochemistry, genetics and molecular biology, computer science, social sciences, pharmacology, toxicology and pharmaceutics, immunology and microbiology, and medicine. The query was further refined by selecting a publication date from January 2020 to June 2022. The data was downloaded on 1 July 2022 and produced an initial selection of 1938 publications. In the current study, we included original articles, review and conference papers, and book chapters published in English. We excluded all of those studies that included comments, editorials, and letters, as well as articles or reviews that were published on preprint websites. The search equation used in this study was:

((ALL(“Omicron” AND “SARS-CoV-2” OR “COVID-19”) AND PUBYEAR > 2020 AND PUBYEAR < 2023) AND (EXCLUDE (DOCTYPE,“le”) OR EXCLUDE (DOCTYPE,“no”) OR EXCLUDE (DOCTYPE,“ed”) OR EXCLUDE (DOCTYPE,“sh”) OR EXCLUDE (DOCTYPE,“er”)) AND ( EXCLUDE (SUBJAREA,“CHEM”) OR EXCLUDE ( SUBJAREA,“ENVI”) OR EXCLUDE (SUBJAREA,“ENGI”) OR EXCLUDE (SUBJAREA,“CENG”) OR EXCLUDE (SUBJAREA,“PHYS”) OR EXCLUDE (SUBJAREA,“AGRI”) OR EXCLUDE (SUBJAREA,“MATE”) OR EXCLUDE (SUBJAREA,“NURS”) OR EXCLUDE (SUBJAREA,“VETE”) OR EXCLUDE (SUBJAREA,“BUSI”) OR EXCLUDE (SUBJAREA,“ECON”) OR EXCLUDE (SUBJAREA,“EART”) OR EXCLUDE (SUBJAREA,“PSYC”) OR EXCLUDE (SUBJAREA,“DECI”) OR EXCLUDE (SUBJAREA,“ENER”) OR EXCLUDE (SUBJAREA,“ARTS”) OR EXCLUDE (SUBJAREA,“DENT”) OR EXCLUDE (SUBJAREA,“MULT”)) AND ( LIMIT-TO (LANGUAGE,“English”) OR EXCLUDE (LANGUAGE,“Spanish”) OR EXCLUDE (LANGUAGE,“Norwegian”) OR EXCLUDE (LANGUAGE,“Portuguese”)) AND ( EXCLUDE (SUBJAREA,“PHAR”) OR EXCLUDE (SUBJAREA,“SOCI”) OR EXCLUDE (SUBJAREA,“NEUR”) OR EXCLUDE (SUBJAREA,“MATH”) OR EXCLUDE (SUBJAREA,“HEAL”)) AND (EXCLUDE (PUBSTAGE,“aip”))).

**Figure 1:**
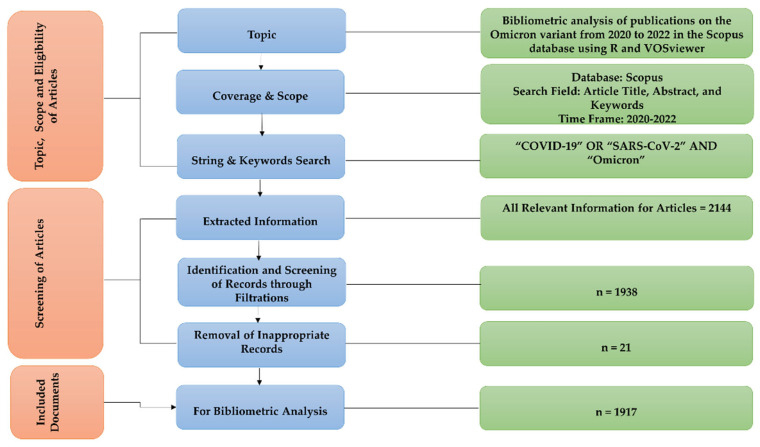
Flow chart of the search approach.

Finally, we reviewed and evaluated all available published data to identify those publications exclusively focused on the Omicron variant and excluded those that referred to Omicron but focused on other variants. Using these inclusion and exclusion criteria, we found a collection of 1917 scientific papers between the earliest accessible date of January 2020 and the latest available date of June 2022. These 1917 records formed the dataset and were the foundation for the bibliometric analysis in this study. Figure 1 shows the search technique used in this study to identify appropriate articles from the Scopus database.

The complete set of bibliographic data was downloaded in .csv format from the Scopus database. Initially, the Bibliometrix R package was installed and loaded with R Studio. The application Biblioshiny was launched by entering Biblioshiny() into the R console. Biblioshiny is a web application that provides access to the Bibliometrix package of R for non-programmers. Bibliometrix provides numerous tools that permit researchers to conduct in-depth bibliometric analysis [42].

Biblioshiny, a statistical software program, was used for data mining in bibliometrics to determine the frequency of the concurrent occurrence of keywords in two scientific articles in order to simplify the intricate keyword network linkages. A .csv Excel file was uploaded to the Biblioshiny interface. Excel files (.csv) and portable network graphics files (.png) were also downloaded and used for data analysis according to the study’s objectives. For the extraction of other patterns, we used VOSviewer to present comprehensive details in Omicron-based research. VOSViewer software, created to generate and visualize bibliometrics maps, was used to study the international publication map. It provides readers the option to develop and visualize a network or relationship by using a text-mining function when quoting an article or problem. It can map more extensive articles and publications using various display options and features, including zooming, scrolling, and searching. The Bibliometrics graphic map can have specific information presented and represented using VOSViewer. Scholars can easily interpret a relationship by displaying a huge bibliometric map, and numerous previous studies have utilized this software in bibliometric analysis [43,44,45].

## 3. Results

### 3.1. Main Information

Table 1 shows the main bibliometric data for the Omicron variant obtained using the Biblioshiny program. A total of 1917 documents were retrieved from 520 sources, and the majority of them were original research articles (1512). The average article citation rate is 3.219.

### 3.2. Annual Publication Growth

**Figure 2:**
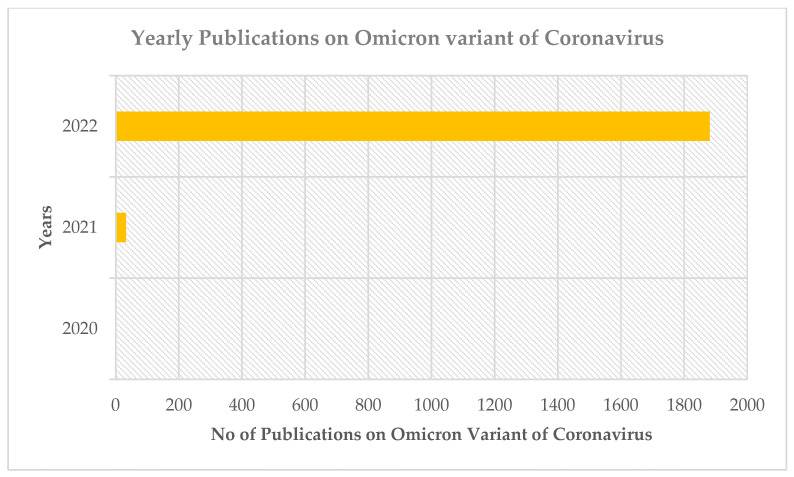
The annual publication growth trend of Omicron variant-based research.

The growth of documents in a bibliometric analysis of the Omicron variant over time is shown in Figure 2. The number of documents is steadily increasing. In 2020, three documents were published, followed by 33 documents in 2021. Furthermore, by 2022, the number of documents on Omicron variant-based research has significantly increased (n = 1881). This demonstrates the tremendous attention scientists worldwide have given the Omicron virus.

### 3.3. The Most Productive and Top Cited Journals

**Figure 3:**
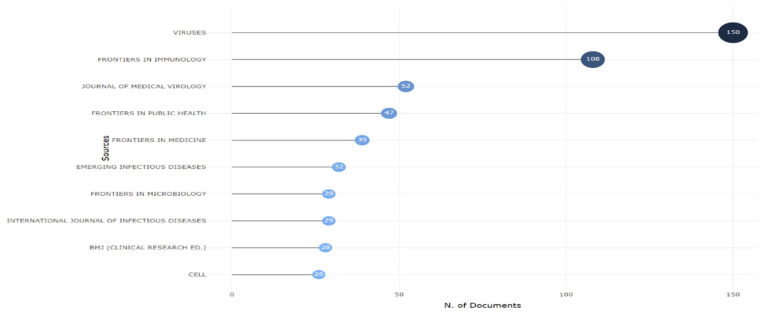
Top ten most productive journals.

The top ten most productive journals for publications on the Omicron variant are shown in Figure 3. The bibliometric analysis for 2020–2022 identified Viruses, with the highest number of publications (150), followed by Frontier in Immunology and the Journal of Medical Virology.

**Figure 4:**
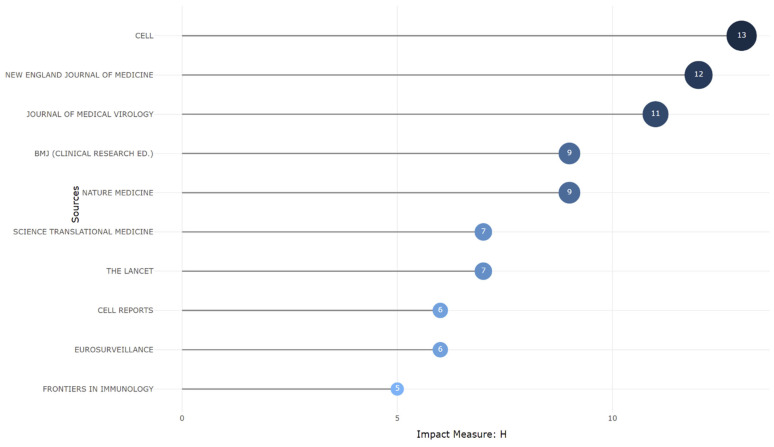
Top ten highly cited journals based on Omicron variant research.

Figure 4 shows the top 10 cited journals that published research on the Omicron variant. The bibliometric analysis identified Cell as the most influential journal worldwide, with the highest h-index (13), g-index (24), and m-index (13). The New England Journal of Medicine was determined to be the second-most highly referenced journal. It is worth noting that newly released journals were also in the top ten. Most of the publications were published in journals with a high impact factor.

### 3.4. The Most Relevant Institutions

**Figure 5:**
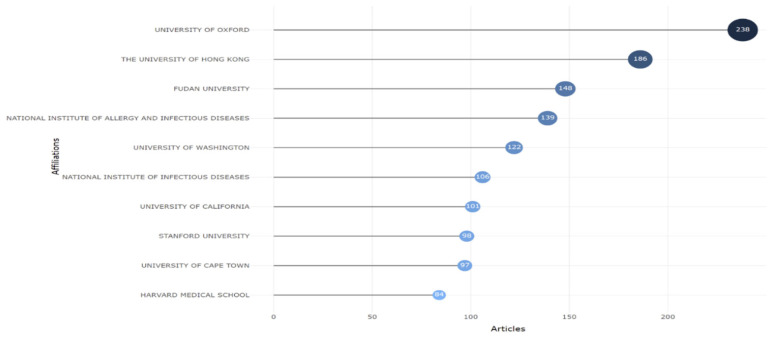
Top ten relevant affiliations contributed to Omicron research.

This study also looked at the publishing output of institutions or authors’ affiliations that contributed to Omicron variant-based research, as shown in Figure 5. The University of Oxford was at the top, with 238 documents.

### 3.5. The Most Relevant Authors

**Figure 6:**
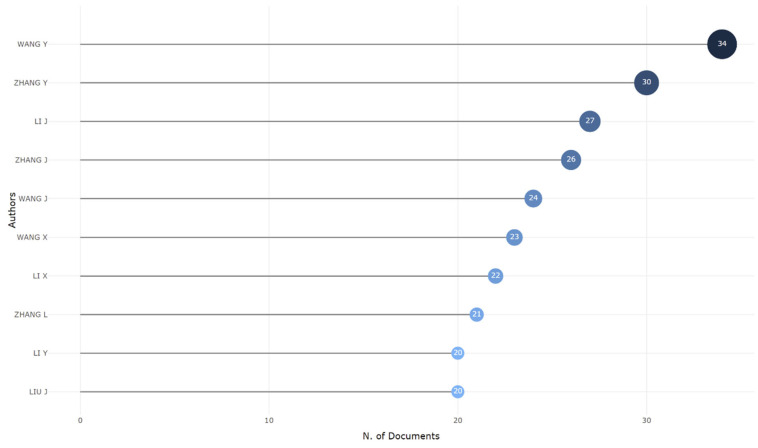
The most relevant authors.

A total of 16,996 authors published 1917 research papers based on the Omicron variant in different publication venues. Wang, Y, was the most productive author, with 34 articles (3.19 fractionalized value) and had the highest frequency peak for average citations per item in 2021, followed by Zhang, Y, (2.65 fractionalized value) and Li, J (2.87 fractionalized value). The top relevant authors are shown in Figure 6.

### 3.6. The Most Relevant Countries by Corresponding Authors

**Figure 7:**
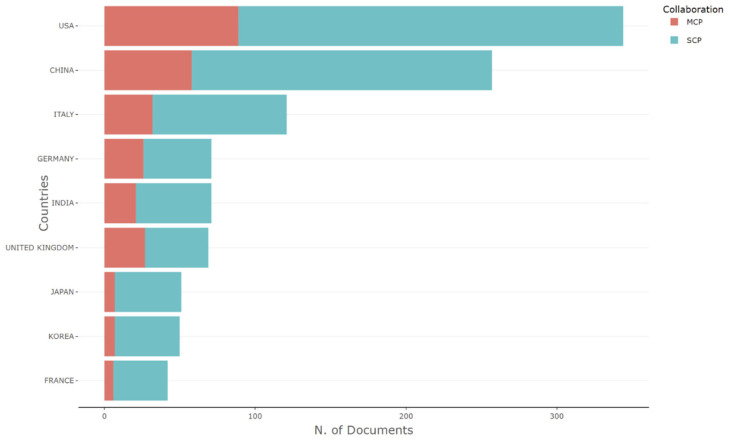
Corresponding authors’ countries representing inter-country (MCP) collaboration and intra-country (SCP) collaboration.

This study also considered publishing output in relation to the corresponding authors’ countries and active participation in Omicron variant-based research. The USA was at the top with 255 single-country publications, 89 multi-country publications, and the highest frequency, 0.179, as shown in Table 2 and Figure 7. The USA, along with China and Italy, leads the world in terms of scientific productivity. These countries lead Scimago Journal & Country Rank’s rankings for global scientific productivity across all disciplines, and in the domain of medicine, this result is not surprising.

### 3.7. The Most Globally Cited Authors

Local citations indicate the frequency with which other documents in the collection have cited an author (or document) in this collection. Local citation score (LCS) and global citation score (GCS) metrics were employed to conduct a more in-depth analysis of the source articles. LCS determined the frequency with which other papers in the collection cited the publications of the authors in the WoS database. Total citations are the number of times the papers in this collection were cited, as defined by GCS. However, the publications referenced were not necessarily in the Omicron variant domain. The higher the LCS, the greater the article’s relevance was to the Omicron variant. The study also used bibliometrics to examine the publishing output of the world’s most-cited authors who participated in Omicron variant-based research. As shown in Table 3, Garcia-Beltran WF ranked first with 210 total citations, 210 citations per year, and a normalized citation score of 70.96. Results also indicate the majority of top-cited papers are about effective vaccination against the Omicron variant.

### 3.8. Frequency Distribution of Scientific Productivity (Lotka’s Law)

**Figure 8:**
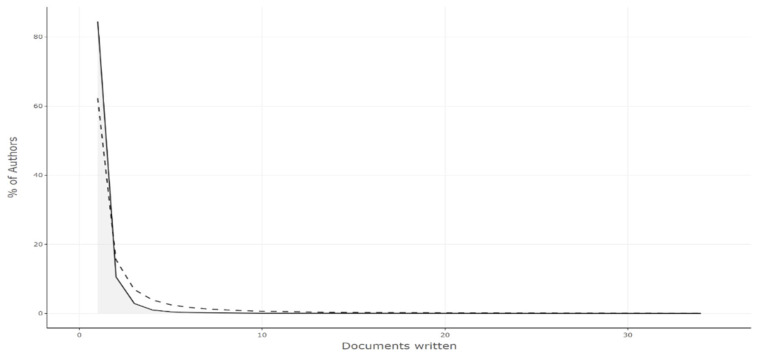
Frequency distribution of scientific productivity (Lotka’s law).

Bibliometric analysis calculates Lotka’s law coefficients for publications on the Omicron variant. Lotka’s law describes the relationship between authors and the number of published papers. In informatics, Lotka’s law describes the distribution of authors over time or within specific subject areas. The distribution of the frequency of authors and the number of publications in the present research field significantly conforms to Lotka’s law shown in Figure 8. The exponent and constant parameters could be influenced by the subject area and its productivity, country, study period, and length. The frequency distribution of scientific productivity according to Lotka’s law is shown in Table 4.

### 3.9. The Most Frequent Words and Relationship with Authors and Countries

**Figure 9:**
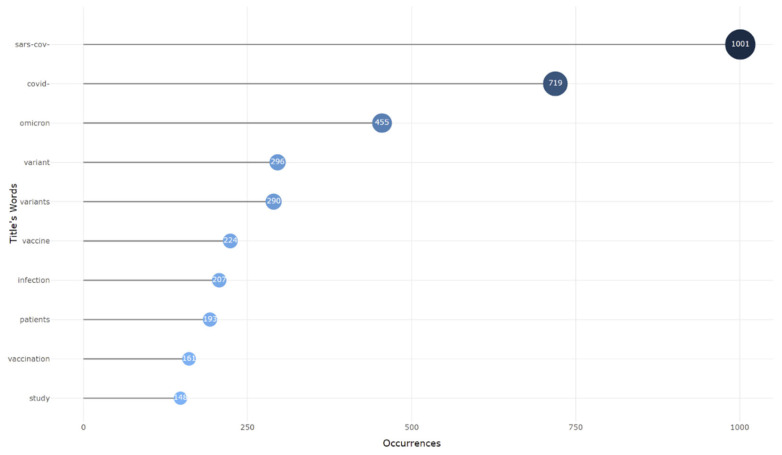
The most relevant words used in Omicron based studies.

**Figure 10:**
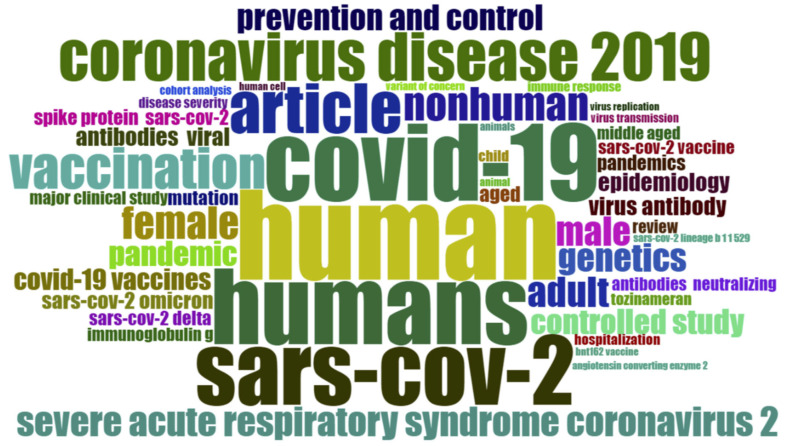
Word cloud of the most frequently used keywords in Omicron-variant-based research.

**Figure 11:**
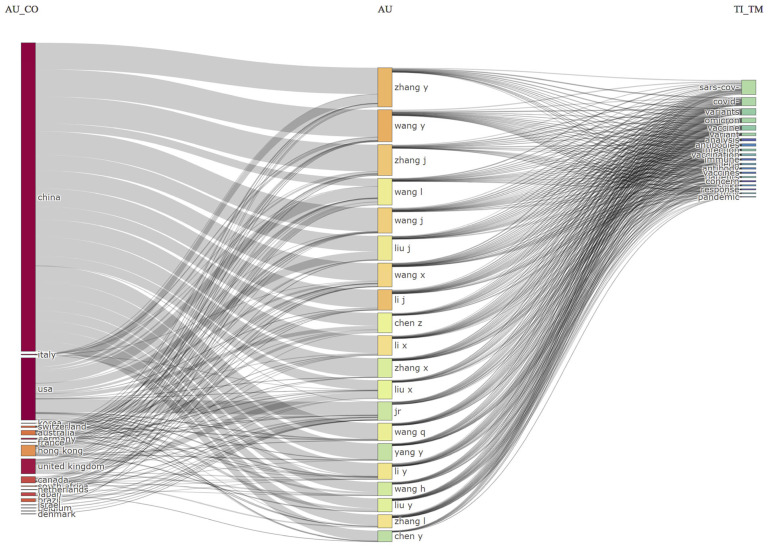
Relationship found in Omicron-based research between countries, authors, and keywords.

“SARS-CoV-2” was the most often used term by authors, with 1001 occurrences, followed by “COVID-19” with 719 occurrences. In Figure 9, the larger the keyword, the more occurrences it has, and vice versa. The annual number of occurrences of all of the main terms increased over time, but some developed faster than others. “Humans,” “COVID-19,” and “SARS-CoV-2,” were the terms with the most significant rise in occurrence, as shown by a word cloud (Figure 10) and a tree map (Figure 11) that presents hierarchical data as a group of nested rectangles. In Figure 11, each group is represented by a rectangle whose area is proportional to the value used for the most frequent words.

The three-field plot, also known as the Sanky plot, showed the relationships between the countries, authors, and keywords of publications on Omicron-based research. Rectangles of various colors were used to depict the relevant elements in the diagram. The value of the sum of the relations originating between the elements that the rectangle represented determined the height of the rectangles (one of the elements: authors, countries, and keywords). A higher rectangle represented the element with the most relations. Sankey diagrams for the top 10 most productive countries and authors are shown in Figure 11, along with their predominant contributions to Omicron-related research. It shows that authors from China, the United States, and the United Kingdom have the most influential research topics on Omicron. The Sankey diagram shows the distribution of the amounts for various items (countries, authors, and keywords). The thickness of the connections (links) indicates a significant flow of information between a set of values.

### 3.10. Collaborative Network of Co-Words in Publications on the Omicron Variant

**Figure 12:**
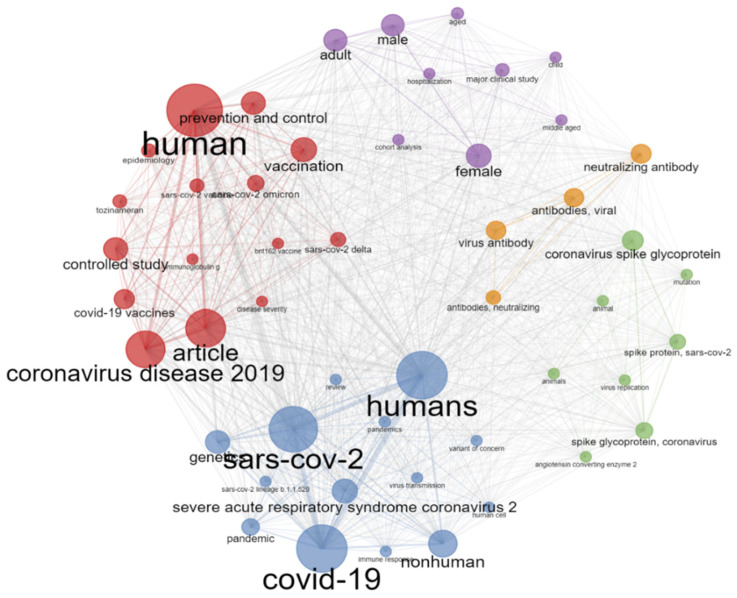
Co-word network visualization: various colors indicate word clusters; label size indicates how frequently each keyword occurs. The same cluster of keywords is frequently listed together.

Figure 12 is a co-word network, a collaborative network of co-words frequently used in studies on the Omicron variant. It shows five clusters colored in red, blue, purple, orange, and green. These five clusters and their nodal positions were based on the values of the measures betweenness, closeness, and page rank. Table 5 shows these measures for each co-word.

### 3.11. Thematic Map

**Figure 13:**
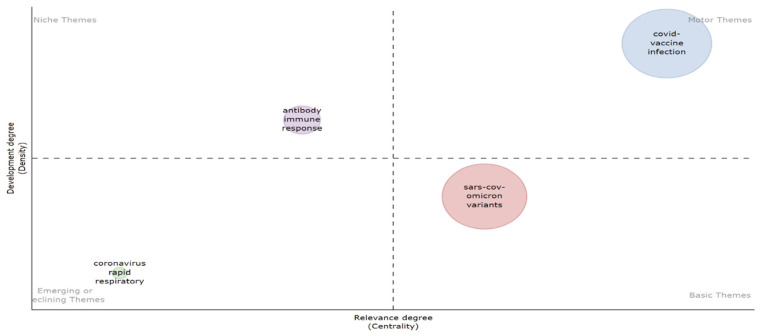
Thematic map representation of keywords in publications on the Omicron variant.

**Figure 14:**
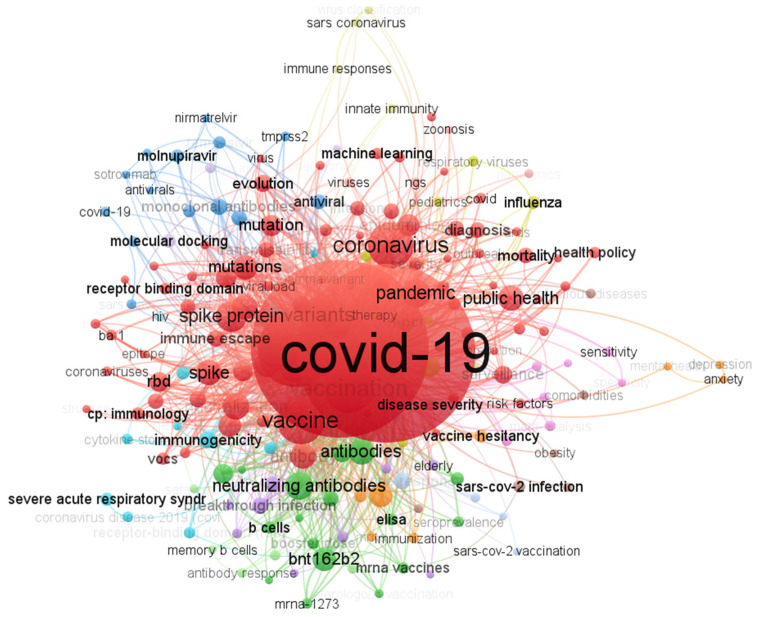
Thematic clusters of keywords in publications on the Omicron variant.

Themes are keyword groupings whose density and centrality can be used to organize them into a single circle and map them as a two-dimensional image. Figure 13 presents a thematic map, which classifies themes according to the quadrant in which they are found, beginning with motor themes in the upper-right quadrant and fundamental themes in the lower-right quadrant. Emerging or disappearing themes are the subject of the lower-left quadrant, and extremely specialized/niche topics are in the upper-left quadrant. The data for keywords in publications on the Omicron variant with their typical measurements are presented in Table 6. Through Callon’s centrality, Callon’s density, rank centrality, and rank density for thematic clusters (Table 6 and Figure 14), theme evolution defined the numerous evolutionary associations that demonstrated field development and the development point, evolutionary routes, and evolutionary drifts of the thematic substance. The primary objective was to recognize and identify relevant topics between 2020 and 2022, such as theme change and progression in Omicron variant research.

### 3.12. Co-Word Analysis: The Conceptual Structure of a Field

The goal of the co-word analysis presented in Table 7 is to use word co-occurrences in a bibliographic collection to map the conceptual structure of a framework [46,47]. It can be performed via dimensionality reduction techniques such as correspondence analysis (CA), multiple correspondence analysis (MCA), and multidimensional scaling (MDS) [20]. For extracting and presenting the most relevant information in a data set, the Factorial Map tool in R can hold the results of CA and MCA from multiple packages. It is worth noting that keyword factorial analysis reveals new information. Unsupervised classification, commonly known as *k*-means clustering, seeks to divide data into meaningful or usable clusters [48,49].

Hierarchical clustering (HC) is a technique that clusters items based on their similarity. The endpoint is a collection of clusters distinct from others and containing similar objects [50]. In bibliometrics, cluster analysis is based on the co-occurrence of two keywords, and a data mining technique is used to divide the complicated keyword network into several smaller clusters. A dendrogram is a tree diagram representing relationships between objects; in HC, it displays the arrangement of clusters formed by the corresponding analysis. Multiple factorial approaches, including CA, MCA, and MDS, can be used to reduce data dimensionality [51]. The *Y*-axis represents the distance measurements of the subjects. Red and blue were used to identify clusters.

Conceptual structure maps are created for a specific scientific field through dimensionality reduction techniques to perform MCA, CA, or MDS to cluster a bipartite network of terms extracted from keyword, title, or abstract fields [50,52]. The more closely the articles’ keywords match, the more closely they are related.

### 3.13. Multiple Correspondence Analysis

**Figure 15:**
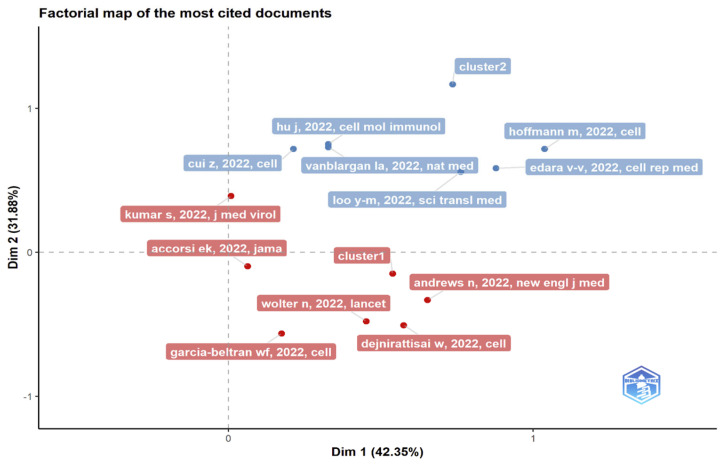
Factorial map using multiple correspondence analysis of the most cited documents related to Omicron-based research.

**Figure 16:**
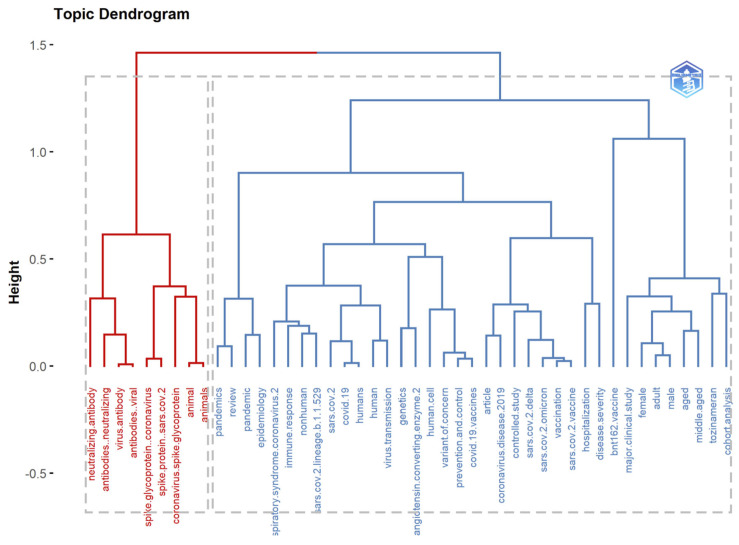
Topic dendrogram using multiple correspondence analysis that shows the hierarchical relationship between topics in the identified field.

**Figure 17:**
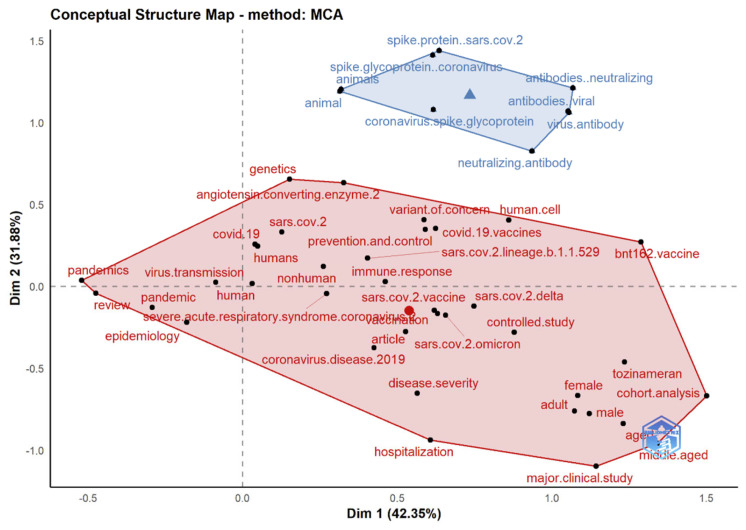
Conceptual structure map (multiple correspondence analysis).

The Conceptual Structure tool in Biblioshiny for Bibliometrix allows the use of MCA to build a conceptual structure of the identified field and *k*-means clustering to find clusters of documents that discuss similar concepts. MCA is a multivariate exploratory technique for graphically and numerically analyzing multivariate categorical data [53]. It investigates the interdependence of a set of categorical variables to discover new latent variables or factors. The relative locations and distribution of dots along the dimensions are used to interpret the results; the closer the words displayed in Figure 15, Figure 16 and Figure 17, the more comparable is their distribution. MCA is a new statistical technique that is gaining popularity in the medical field. This method reduces the number of dimensions in data, resulting in two-dimensional visualizations that show the similarities between data. The terms that are closer to the map’s center and more widely diffused in this study are those that have received more attention in recent years, while those that are more evenly distributed are associated with less-often-discussed research topics.

### 3.14. Correspondence Analysis

**Figure 18:**
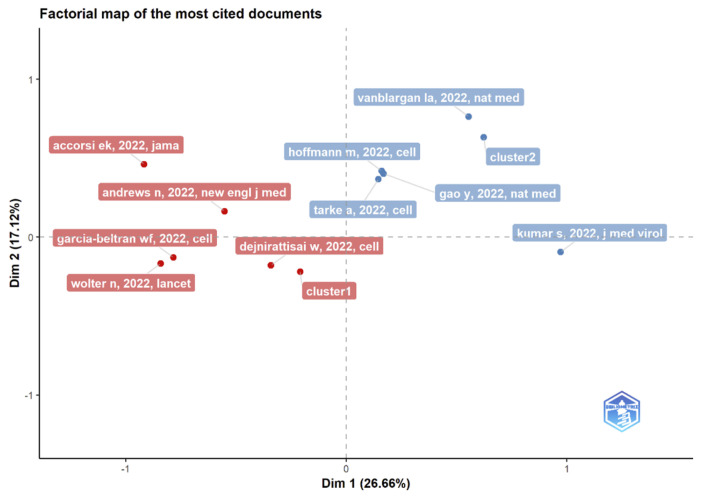
Factorial map using a correspondence analysis of the most cited documents related to Omicron-based research).

**Figure 19:**
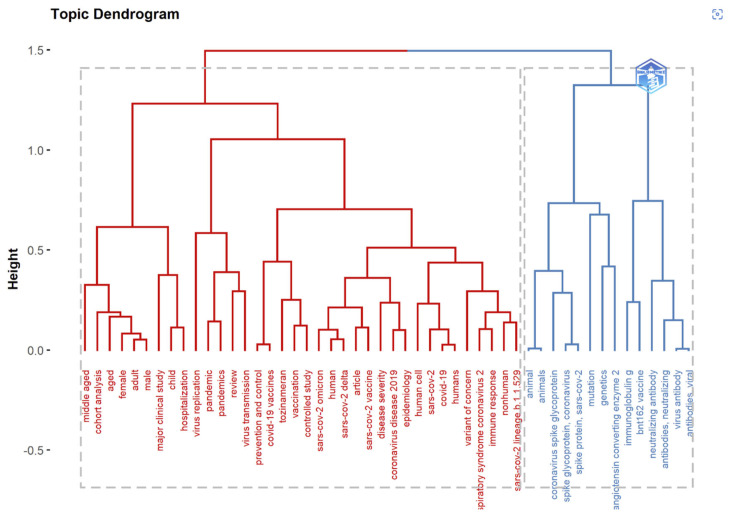
Topic dendrogram using correspondence analysis that shows the hierarchical relationship between the topics in the identified field.

**Figure 20:**
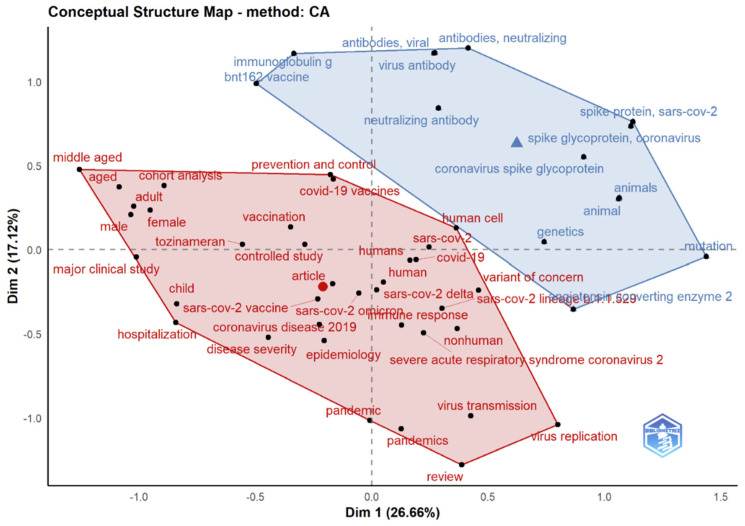
Conceptual structure map using correspondence analysis that integrates and correlates the knowledge of current studies on Omicron.

Correspondence analysis is a graphical method of understanding the relationship between variables in a contingency table. It is an extension of principal component analysis and is designed to evaluate links between qualitative variables (or categorical data). It provides a technique for summarizing and visualizing data sets using two-dimensional graphs [54]. It gives factor scores (coordinates) to row and column points of the contingency table. The link between the row and column components in the contingency table is visualized graphically using these coordinates. A common question when considering a two-way contingency table is whether certain row constituents are connected to specific column constituents. CA is used to represent the rows and columns of a two-way contingency table as points in a low-dimensional space. The placements of row and column points correspond to their table relationships. As indicated in Figure 18, Figure 19 and Figure 20, the goal is to obtain a global picture of the data, which may be used for interpretation.

### 3.15. Multidimensional Scaling

**Figure 21:**
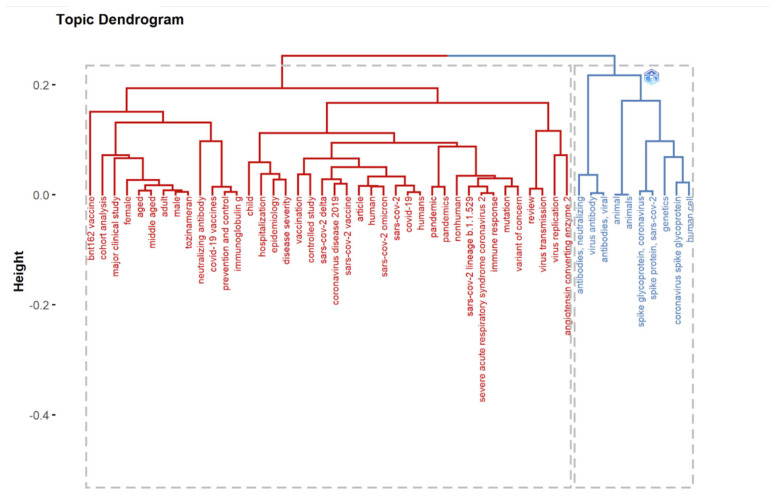
Topic dendrogram using multidimensional scaling that shows the hierarchical relationship between the topics in the identified field.

**Figure 22:**
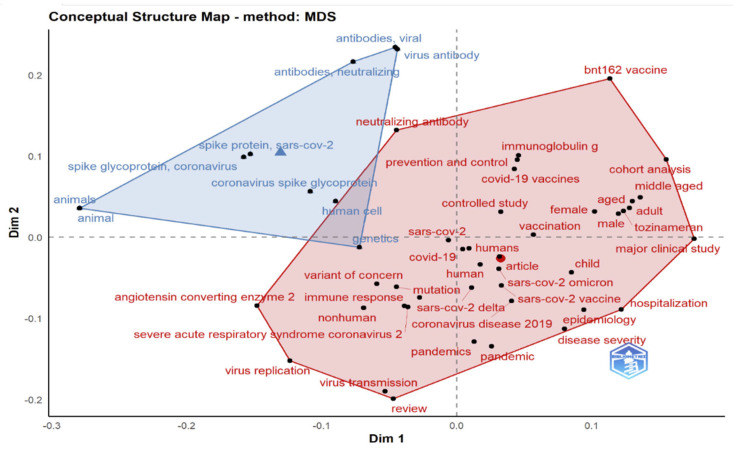
Conceptual structure map using multidimensional scaling that integrates and correlates the knowledge of current studies on Omicron.

Multidimensional scaling is a method of multivariate data analysis for visualizing sample similarity and dissimilarity by plotting points in two-dimensional plots, as shown in Figure 21 and Figure 22. MDS offers the best solution for representing data in a lower-dimensional space, wherein k is the number of dimensions [55]. The dissimilarity matrix represents the distances between pairs of objects and is fed into an MDS algorithm as input data. The input in MDS is the dissimilarity matrix that represents the distances between entities.

## 4. Discussion

Numerous inferences and implications have been drawn from bibliometric and content analyses, which have been the subject of extensive discussion. The growth of the scientific output on the Omicron variant of SARS-CoV-2 and related topics reached a peak in the first quarter of 2022, with a dramatic increase in the number of publications in 2022. Researchers from all around the world have undertaken several studies (research papers, systematic reviews, and meta-analyses) on the diagnosis, treatment, management, and prevention of Omicron. However, relatively few studies have been conducted that specifically analyses bibliometric data on it despite the significance of bibliometric studies as a tool for examining research quantity, directions, and interactions between academics and medical practitioners.

According to the tabulated statistics, the majority of the documents were co-written, with only 7.6% being single-authored, which indicates a high rate of collaboration on this topic. The Sankey plots using three primary metadata fields provide valuable insights based on the relationship between domains such as the authors linking their work to a particular keyword and the countries participating in this research area; for example, author Wang, Y, from China had the most significant impact on Omicron variant research. This study also identifies the most influential authors in the field and their most productive years; the majority of authors were more prolific after 2020. Wang, Y; Zhang, Y; and Li, J, were the only authors to have published their work consistently. Our results also indicate a strong relationship of China, the United States, and Italy with the research on Omicron variants.

The results of our study indicate that there are five clusters for keywords, and each cluster contains more than four words. Cluster one contains more than 11 words, mainly focusing on SARS-CoV-2 Omicron vaccination, prevention, and treatment, reflecting the significance of controlling emerging Omicron variants. Fourteen keywords are present in cluster two, focusing on the evolution of variants of concerns after the pandemic of COVID-19. Due to the continuous evolution of SARS-CoV-2, it is a hot topic in research. Cluster three contains eight keywords that mainly focus on the virus mutation sites and virus replication in animals. Cluster four contain words like viral antibodies and neutralizing antibody, mainly focusing on the diagnosis of the virus. Interestingly, cluster five includes adults, age, male, and female, indicating studies on different population groups to see the clinical severity with age and gender.

Scientific maps use knowledge frameworks and describe a research domain’s structural and dynamic elements. In this study, they were used to provide a comprehensive overview of the significant trends and findings in Omicron-variant-related research in the form of conceptual structures, which defined the major themes, topics, and intellectual structures that categorized how an author’s work impacted this research community. The study of the development of concepts or situations across time could be another of its beneficial applications. This methodology offers researchers with the most prominent publications for each theme cluster, which can be used to restrict investigations pertinent to a specific theme. The thematic map developed by the clustering method can provide information on the significance of the topics based on centrality and density, which allows forecasts of the future expansion of the themes.

This study provides an overview of the current state and trends in Omicron variant research. Due to the topic’s multidisciplinary nature and despite the extraordinary efforts of the scientific community to produce a large number of studies to address the issue, worldwide characterization of the various domains is required. Numerous bibliometric studies have focused on COVID-19, but its evolving variants have been identified as mitigating factors for COVID-19-related research. This study is one of the first bibliometric investigations of publications on the Omicron variant to identify the most prolific authors, reference articles, institutions, countries, and fields of study in the Scopus database. As the pandemic progresses, a shift can be observed in the research emphasis of the studies evaluated.

In general, there are substantial differences between studies published in 2020 and those issued the following year. The trend in the subject matter covered in the scientific literature parallels the global spread of the Omicron variant and the related communication efforts. The terms fear, anxiety, mental health, awareness, resiliency, and lockdown of 2020 were replaced by behavior, immunization, risk perception, social distancing, health promotion, and telemedicine in 2021. The references to the most influential studies published in academic journals indexed by the Scopus database are among the data obtained for authorship analysis in this study.

The results of the bibliometric analysis indicate that there is a limited number of authors producing the most influential works. Most publications are open access, resulting in the rapid and widespread dissemination of contributions and the emergence of numerous authors as the field develops. In addition, it is essential to note that the number of citations is growing, which indicates the current significance of the topic. Consistent with previous research, the USA, along with China and Italy, leads scientific production in this area. This outcome is not unexpected given that these countries are at the top of Scimago Journal & Country Rank’s rankings for the world’s scientific productivity across all disciplines and in the medicine category [56]. The data reveal a variety of methodologies and specialties, even among the most prolific researchers, illustrating the interdisciplinarity of the research. The effectiveness and scope of journals are key factors in the transmission of knowledge to all stakeholders. According to our research, “Viruses” and “Frontiers in Immunology” had the most publications on the topic.

Results in Figure 17, Figure 20 and Figure 22 indicate a conceptual structure map of multiple correspondence analysis, correspondence analysis, and multidimensional scaling analysis. It reveals a factorial analysis of our data, presenting a classification of common keywords from all data records in two classifications. The classification in blue represents keywords like an animal, spike glycoprotein, etc. The classification in red represents more specific keywords, like a variant of concern, omicron, genetics etc.

COVID-19 may have resulted in the largest concentration of scientific resources ever seen. Despite the availability of resources, such as repositories, and their importance, journal articles remain the main disseminators of scientific knowledge and discovery. This study has significant advantages over earlier work that focused simply on systematic reviews. To our knowledge, this is the first bibliometric analysis of the literature on the Omicron variant of SARS-CoV-2 published between 2020 and 2022. Examination of the quality of the listed publications and publishers is another strength of this bibliometric study. The aim of this study was limited to publications that were indexed in the Scopus database and related to the Omicron variant. Although a comparison between the datasets of different databases is outside the scope of this investigation but it may return distinct sets of entries when searched, and results can vary with this analysis.

## 5. Conclusions

This study examined and evaluated the global scientific output in research on the Omicron variant by analyzing records from the Scopus database and identified the current top researchers, mapping their regional distribution and publications. Notably, the majority of journals that have published research on the pandemic have implemented open-access policies to facilitate the sharing of their resources, thereby accelerating the spread of scientific knowledge. USA, Italy, and China have the largest number of citations. Wang, Y, is the most prolific author found in the bibliometric examination of publications on Omicron. The journal with the highest preference for publications on Omicron is Viruses. SARS-CoV-2 and “Omicron” are the top keywords, used by authors 978 and 408 times, respectively. The Biblioshiny application from R’s Bibliometrix package and VOSviewer provided significant study streams and topics.

We expect that, by comprehensively summarizing the patterns in Omicron-related research, our findings will provide valuable insight into future research paths and perspectives in the rapidly evolving field of COVID-19. Numerous opportunities for significant future work exist.

## 6. Future Directions

Future research should investigate numerous more unique and advanced ML-based technologies for bibliometric analysis, including the estimation of the topic dominance based on ranking to produce cluster prediction. This study focused on the Scopus database, although other databases such as Dimensions, Web of Science, Cochrane Library, and PubMed can also be utilized. Another possible future route is designing and developing a visualization tool that covers more dimensions and provides more data on the initial shot.

## Figures and Tables

**Table 1:** Main information.

Description	Results
**Key Information About Data**	
Time Span	2020:2022
Sources (Journals, Books, etc.)	520
Documents	1917
Average Number of Citations per Document	3.219
References	100,743
**Types of Documents**	
Article	1512
Review	393
Conference Paper	7
Book Chapter	4
Book	1
**Contents of Documents**	
Plus Keywords (Id)	7267
Keywords Used by Authors (DE)	3705
**Authors**	
Single-Authored Document Authors	74
Multi-Authored Document Authors	1843
**Collaboration among Authors**	
Single-authored Docs	101
Co-Authors per Doc	11.3
International Co-authorships%	28.22

**Table 2:** Corresponding authors’ countries.

Country	Articles	Frequency	Single-Country Publication	Multiple-Country Publication	Multiple-Country publication Ratio
USA	344	255	89	0.179	0.259
China	257	199	58	0.134	0.226
Italy	121	89	32	0.063	0.264
Germany	71	45	26	0.037	0.366
India	71	50	21	0.037	0.296
United Kingdom	69	42	27	0.036	0.391
Japan	51	44	7	0.027	0.137
Korea	50	43	7	0.026	0.14
France	42	36	6	0.022	0.143

**Table 3:** Top ten globally cited authors.

Paper	Title	Total Citations	Total Citations per Year	Normalized Citation Score
Garcia-Beltran WF, 2022, Cell	mRNA-based COVID-19 vaccine boosters induce neutralizing immunity against SARS-CoV-2 Omicron variant	210	210.00	70.96
Andrews N, 2022, New Engl J Med	COVID-19 Vaccine Effectiveness against the Omicron (B.1.1.529) Variant	165	165.00	55.75
Hoffmann M, 2022, Cell	The Omicron variant is highly resistant against antibody-mediated neutralization: Implications for control of the COVID-19 pandemic	160	160.00	54.06
Wolter N, 2022, Lancet	Early assessment of the clinical severity of the SARS-CoV-2 omicron variant in South Africa: a data linkage study	145	145.00	48.99
Dejnirattisai W, 2022, Cell	SARS-CoV-2 Omicron-B.1.1.529 leads to widespread escape from neutralizing antibody responses	137	137.00	46.29
Vanblargan La, 2022, Nat Med	An infectious SARS-CoV-2 B.1.1.529 Omicron virus escapes neutralization by therapeutic monoclonal antibodies	124	124.00	41.90
Torjesen I, 2021, BMJ	SARS-CoV-2 Omicron-B.1.1.529 leads to widespread escape from neutralizing antibody responses	88	44.00	4.98
Kumar S, 2022, J Med Virol	An infectious SARS-CoV-2 B.1.1.529 Omicron virus escapes neutralization by therapeutic monoclonal antibodies	87	87.00	29.40
Accorsi EK, 2022, JAMA	COVID-19: Omicron may be more transmissible than other variants and partly resistant to existing vaccines, scientists fear	87	87.00	29.40
Tarke A, 2022, Cell	SARS-CoV-2 vaccination induces immunological T cell memory able to cross-recognize variants from Alpha to Omicron	85	85.00	28.72

**Table 4:** Frequency distribution of scientific productivity according to Lotka’s law.

Documents Written	Number of Authors	Proportion of Authors
1	14,346	0.844
2	1790	0.105
3	483	0.028
4	163	0.01
5	72	0.004
6	41	0.002
7	30	0.002
8	13	0.001
9	13	0.001
10	9	0.001

**Table 5:** Co-word network analysis for Omicron-based research.

Node	Cluster	Betweenness	Closeness	Page Rank
Human	1	1.785	0.020	0.060
Article	1	0.724	0.020	0.041
Coronavirus Disease 2019	1	1.028	0.020	0.040
Vaccination	1	0.276	0.020	0.026
Prevention and Control	1	0.175	0.020	0.024
Controlled Study	1	0.281	0.020	0.024
COVID-19 Vaccines	1	0.126	0.020	0.021
Epidemiology	1	0.029	0.020	0.015
SARS-CoV-2 Omicron	1	0.079	0.020	0.017
SARS-CoV-2 Delta	1	0.107	0.020	0.016

**Table 6:** Thematic cluster measurements for keywords in publications on the Omicron variant.

Clusters	Callon’s Centrality	Callon’s Density	Rank Centrality	Rank Density	Cluster Frequency
SARS-CoV-2	2.418954907	7.849414925	3	2	4094
COVID-19	2.55908645	9.615896545	4	4	4648
Coronavirus	0.988808353	7.029991359	1	1	660
Antibody	1.53171894	8.060157706	2	3	1169

**Table 7:** Co-word analysis by clustering.

Words	Dimension-1	Dimension-2	Cluster
Humans	−0.01	−0.05	1
SARS-CoV-2	−0.01	−0.05	1
COVID-19	−0.01	−0.05	1
COVID-19 vaccines	0.03	−0.09	1
Mutation	−0.02	−0.08	1
Spike glycoprotein, coronavirus	−0.01	-0.09	1
Pandemics	0.04	−0.02	1
Vaccination	0	−0.1	1
Adult	−0.08	0.21	1
Middle aged	−0.18	0.14	1

## Data Availability

The data presented in this study are available on request from the corresponding author.
